# The Man‐PTS subunit ⅡC is responsible for the sensitivity of *Listeria monocytogenes* to durancin GL

**DOI:** 10.1002/fsn3.1285

**Published:** 2019-12-05

**Authors:** Xueyou Wu, Xingrong Ju, Lihui Du, Lifeng Wang, Rong He, Zhengxing Chen

**Affiliations:** ^1^ School of Food Science and Technology Jiangnan University Wuxi China; ^2^ College of Food Science and Engineering Nanjing University of Finance and Economics Nanjing China

**Keywords:** bacteriocin, durancin GL, *Listeria monocytogenes*, target cell recognition

## Abstract

Target cell recognition is an important issue in the realization of bacteriocin's activity. In this report, we provide genetic and biochemical evidence of durancin GL, a new bacteriocin produced by *Enterococcus durans* 41D, and use ⅡC subunit in the mannose phosphotransferase system (Man‐PTS) of *Listeria monocytogenes* as target/receptor. First, the *L. monocytogenes* mutants with Man‐PTS IIC or IID deletion were constructed with the vector pHoss1. Then, the utilization of glucose and mannose and the sensitivity to durancin GL of the mutant strains were investigated. Afterward, the interactions between durancin GL and the subunits of IIC or IID in Man‐PTS of *L. monocytogenes* were characterized by yeast two‐hybrid system. The results showed that the *L. monocytogenes* mutants with either IIC or IID deletion were not only resistant to durancin GL, but also their absorption and utilization of glucose and mannose were not disturbed by the presence of durancin GL. Finally, in situ detection of the interaction between durancin GL and Man‐PTS subunits of IIC or IID by yeast two‐hybrid system revealed that there was a strong interaction between durancin GL and Man‐PTS subunit IIC. However, the interaction between durancin GL and Man‐PTS subunit IID was not present or weak. Based on the experimental evidence above, the Man‐PTS subunit IIC is responsible for the sensitivity of *L. monocytogenes* to bacteriocin durancin GL.

## Graphical abstract

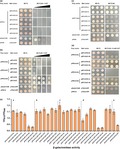

## 1 INTRODUCTION

It is of great importance to control the foodborne pathogen *Listeria monocytogenes* (*L. monocytogenes*) on food safety perspective, and also for human health (Gray & Killinger, 1966; Lebreton, Stavru, & Cossart, 2015; Radoshevich & Cossart, 2018; Swaminathan & Gerner‐Smidt, 2007). *L. monocytogenes*, a member of the genus *Listeria*, is widely distributed in agricultural environments, such as soil, manure, water, and plants (Gandhi & Chikindas, 2007; Smith, Moorhouse, Monaghan, Taylor, & Singleton, 2018). Foods like milk (Verraes et al., 2015), meat products (Alvarez Ordonez, Leong, Hickey, Beaufort, & Jordan, 2015), vegetables (Valimaa, Tilsala‐Timisjarvi, & Virtanen, 2015), processed ready‐to‐eat and cold‐stored meat, and dairy products are considered high‐risk foods for *L. monocytogenes* infections (Tompkin, 2002). This organism is a recognized foodborne pathogen that causes many diseases, from mild gastroenteritis to severe blood and/or central nervous system infections, as well as abortion in pregnant women (Vazquez‐Boland et al., 2001). The lethal rate of listeriosis is more than 25% (Pamer, 2004). In addition, *L. monocytogenes* was recently reported to have the ability to form biofilm, which brought a bigger problem to food processing (Giaouris et al., 2015; Mathur et al., 2018). Therefore, it is of vital practical significance to inhibit the growth of *Listeria* and control its contamination in foods to ensure the quality and safety of food.

Bacteriocins are antimicrobial proteins or polypeptides synthesized by bacterial ribosomes and secreted to extracellular (Bastos, Coelho, & Santos, 2015; Vanderwal, Luirink, & Oudega, 1995). Recent studies have shown that bacteriocin can be degraded in human body, nontoxic, and target bacteria are not easy to produce resistance (Franz et al., 2018; Mathur et al., 2017). Besides nisin, bacteriocins produced by lactic acid bacteria (LAB) are attracting considerable interest for use as alternative food preservatives (Favaro, Penna, & Todorov, 2015), especially the class IIa bacteriocins that is well recognized for their high antilisterial activity. However, the target cell recognition mechanism of class IIa bacteriocins is poorly understood.

Previous research suggested that mannose phosphotransferase system (Man‐PTS) functions in the regulation of various bacterial physiological processes (Postma, Lengeler, & Jacobson, 1993; Reizer et al., 1988; Saier, 1989). The Man‐PTS is consisted by four components named IIA, IIB, IIC, and IID. It was reported that there was a putative Man‐PTS IIAB component in a leucocin A‐resistant strain of *L. monocytogenes* (Ramnath, Beukes, Tamura, & Hastings, 2000). Also, interruption of either the proximal (mptA) or distal (mptD) gene in *Enterococcus faecalis* resulted in resistant to mesentericin Y105 (Karine et al., 2001). Thus, the subclass IIa bacteriocin is likely to use a Man‐PTS component as specific target (Hechard & Sahl, 2002). Furthermore, sensitivity to class IIa bacteriocins of lactic acid bacteria was recently associated with Man‐PTS permease in *L. monocytogenes*, and it shows that expression of mptC alone is sufficient to confer sensitivity to class IIa bacteriocins in *Lactococcus lactis* (Ramnath, Arous, Gravesen, Hastings, & Hechard, 2004). The antibacterial mechanism of lactococcin A, a class Ⅱa bacteriocin, on target bacteria was described in detail by Diep, Skaugen, Salehian, Holo, and Nes, (2007). Kjos, Nes, and Diep, (2009) pointed that the level of bacteriocin susceptibility for a bacterial is primarily determined by differences in its Man‐PTS proteins, although the expression levels of the corresponding genes also play an important role. The results of pediocin PA‐1 (Kjos, Nes, & Diep, 2011; Opsata, Nes, & Holo, 2010; Zhou, Wang, Wang, Ren, & Hao, 2016), pediocin‐like bacteriocin (Colombo, Chalon, Navarro, & Bellomio, 2018), and garvicins A, B, and C also support this conclusion.

Durancin GL, a new bacteriocin produced by *Enterococcus durans* 41D, was found to have high antilisterial activity (Du, Somkuti, Renye, & Huo, 2012). By alanine‐scanning mutational analysis with site‐directed mutagenesis, it showed that durancin GL residues were important for antimicrobial activity and specificity, such as three mutations lost their antimicrobial activity, 10 mutations demonstrated a decreased effect on the activity, and seven mutations exhibited relatively high activity; besides, four mutants demonstrated a narrower antimicrobial spectrum than wild‐type durancin GL, and another four mutants displayed a broader target cell spectrum and increased potency relative to wild‐type durancin GL (Ju et al., 2015). Study on phenotypic and genotypic alterations of durancin GL‐resistant enterococcus durans strains showed that durancin GL can cause damage to bacterial cells of wild bacteria and the increased unsaturated fatty acid and decreased mannose phosphotransferase system gene expression in resistant strains could contribute to durancin GL resistance (Du, Liu, Liu, Ju, & Yuan, 2016). These findings broaden our understanding antimicrobial activity of durancin GL; however, the related mechanism is not clear yet, and this study focused on clarifying the targeting inhibition of *L. monocytogenes* by durancin GL.

## 2 MATERIALS AND METHODS

### 2.1 Bacterial strain and culture condition

Bacterial strains, plasmids, and primers used in this study are listed in Table 1. *L. monocytogenes* Scott A was provided by Jiangsu provincial center for disease control and prevention in Nanjing, China. *L. monocytogenes* was propagated in brain heart infusion (BHI) medium (Land Bridge Co. Ltd) under aerobic conditions at 37°C and 200 rpm in a shaking incubator. A single colony of yeast NMY51 was inoculated in yeast extract peptone dextrose (YPD) liquid medium (Land Bridge Co. Ltd) and cultured in an oscillator at 30°C and 200 rpm. The growth of *L. monocytogenes* and yeast NMY51 was spectrophotometrically measured with optical density (OD) at 600 nm (UV–Vis spectrophotometer, U‐3900; Hitachi).

**Table 1:** Strains, plasmids, and primers used in this study

Strain, plasmid, or primer	Description or sequence (5′→3′)	Source
Strain		
*Escherichia coli* Top10	Chemically competent cell	Takara Bio Company
*E. coli* DH5α	Chemically competent cell	Takara Bio Company
*E. coli* Rosetta (DE3)	Durancin GL expression strain	TransGen Biotech Co., Ltd
*Listeria monocytogenes* Scott A	Indicator bacteria	Our laboratory
Yeast NMY51	Yeast reporter strain, chemically competent cell MATa his 3Δ200 trp1−901 leu 2–3,112 ade2 LYS 2::(lex Aop )_4_‐HIS3 ura3::(lexAop )_8_‐lac Z ade2::(lexAop)_8_‐ADE2GAL4	Dualsystems Biotech
Plasmid		
pUC57	Clone vector	Takara Bio Company
pHoss1	Allelic replacement vector	Biovector NTCC Inc.
pGEX/his‐durAB	Durancin GL expression vector	Our laboratory
pPR3‐N	Prey vector	Dualsystems Biotech
pBT3‐STE	Bait vector	Dualsystems Biotech
pBT3‐SUC	Bait vector	Dualsystems Biotech
pNubG‐Fe65	Positive control prey vector	Dualsystems Biotech
pOst1‐NubⅠ	Positive control prey vector	Dualsystems Biotech
pTSU2‐APP	Positive control bait vector	Dualsystems Biotech
Primer		
Pr01	ACGCGTCGACGAGGGAAAAAGATGGTAGGAATTAT	
Pr02	CCATTCTATTCTCCTCCTTTTTTTAAATAAACCTCCTATTTTAATTTTTT	
Pr03	TAAAAAATTAAAATAGGAGGTTTATTTAAAAAAAGGAGGAGAATAGAATGGC	
Pr04	GAAGATCTCATATATCTAAACAAAAGAGGCTCG	
Pr05	CATGCCATGGGGAAAGATGATGTTGAAAC	
Pr06	CCAGCCTCTTTTCGATCAGCTTATTTTCTATTCTCCTCCTTTTTTTATTA	
Pr07	TATTAATAAAAAAAGGAGGAGAATAGAAAATAAGCTGATCGAAAAGAGGC	
Pr08	GAAGATCTTAAAATCTTCTCCATTTTCTTCCC	
Pr09	AAGGCCATTACGGCCGCAACTTATTATGGAAATGGTGT	
Pr10	CCGGCCGAGGCGGCCCTATCTAGGAGCCCAAGGTCCAT	
Pr11	CCGGCCGAGGCGGCCCCTCTAGGAGCCCAAGGTCCAT	
Pr12	AAGGCCATTACGGCCATGTCTGTCATATCAATAATTTTAG	
Pr13	CCGGCCGAGGCGGCCTTAATAGTCGTTTAATATATCGCCCA	
Pr14	CCGGCCGAGGCGGCCCCATAGTCGTTTAATATATCGCCCA	
Pr15	AAGGCCATTACGGCCATGGCAGAAAAAATCGAATTATC	
Pr16	CCGGCCGAGGCGGCCTTACAGAAGCCCGATTAAGTGAC	
Pr17	CCGGCCGAGGCGGCCCCCAGAAGCCCGATTAAGTGAC	

### 2.2 Effect of durancin GL on *Listeria monocytogenes* growth curve

Durancin GL was prepared according to the method of Ju et al., (2015) Bacteriocin titer was determined according to the literature (Van Reenen, Dicks, & Chikindas, 1998), and the unit of bacteriocin titer is AU/ml. *L. monocytogenes* Scott A was inoculated into BHI liquid medium with 1% (v/v) inoculation volume. A final concentration of durancin GL at 100 AU/ml or 200 AU/ml was added to the BHI culture when *L. monocytogenes* was in logarithmic growth period. The growth of *L. monocytogenes* was spectrophotometrically measured. According to the literature (Andrews, 2001), the live bacteria in 100 μl culture above were enumerated on BHI agar plate after serial dilution.

### 2.3 Effect of durancin GL on carbohydrate utilization by *Listeria monocytogenes*

According to Premaratne, Lin, & Johnson, (1991), the basic medium with the lowest requirement for the growth of *L. monocytogenes* was prepared, and the only carbohydrates used in the basic medium above were glucose, fructose, mannose, cellobiose, trehalose, and maltose, respectively. All the compounded carbohydrate medium was sterilized at 121°C for 15 min. After the medium was cooled to room temperature, a certain concentration of durancin GL was added in with a final concentration of 0 AU/ml (as control), 50 AU/ml, 100 AU/ml, and 200 AU/ml, respectively. Then, the growth of *L. monocytogenes* was followed by OD_600_ value.

### 2.4 Gene knockout box construction

According to the manufacturer's protocol of bacterial genome extraction kit (Sangon Biotech), the genomic DNA in *L. monocytogenes* was extracted by SZ‐10 column silica gel membrane. Man‐PTS ⅡC knockout box was constructed with primer pairs Pr01 (containing *Sal* I restriction site)/Pr02 and Pr03/Pr04 (containing *Bgl* Ⅱ restriction site), and Man‐PTS ⅡD knockout box was constructed with primer pairs Pr05 (containing *Nco* I restriction site)/Pr06 and Pr07/Pr08 (containing *Bgl* Ⅱ restriction site) in Table 1. The constructed Man‐PTS ⅡC or ⅡD knockout box was digested overnight by *Sal* I/*Bgl* Ⅱ enzymes or *Nco* Ⅰ/*Bgl* Ⅱ enzymes, respectively. Then, they were linked to the pHoss1 vector digested by the same enzymes. All the restriction enzymes were provided by Thermo Fisher Scientific (China) Co., Ltd. The recombinant knockout vectors were transformed into *Escherichia coli* DH5α competent cells (TransGen Biotech Co., Ltd), and the sequence was verified by sequencing in Sangon Biotech Co., Ltd.

### 2.5 Screening of *Listeria monocytogenes* gene mutants

Referring to the optimized method by Park & Stewart, (1990), *L. monocytogenes* cells were treated by penicillin (the final concentration was 10 μg/ml, Sigma‐Aldrich LLC). With a ratio of 20 μg plasmid DNA per mL *L. monocytogenes* Scott A (1 × 10^10^ CFU/ml), the recombinant vectors were transformed into *L. monocytogenes* by electroporation at a field strength of 10 kV/cm (pulse duration 5 ms). The cells were plated on BHI agar plates containing penicillin (the final concentration was 50 μg/ml) to screen for the transformants. Single colonies on the plates were selected for PCR verification and then sent to Sangon Biotech Co., Ltd for sequence verification. The Man‐PTS ⅡC and ⅡD gene mutant strains were designated *L. monocytogenes* GKC and *L. monocytogenes* GKD, respectively.

### 2.6 Characterization of *Listeria monocytogenes* gene mutants

The sensitivity to durancin GL of *L. monocytogenes* mutants was tested on BHI agar plates with reference to the literature (Seegal & Holden, 1945). The concentration of durancin GL in the test was 50 AU/ml, 100 AU/ml, and 200 AU/ml, respectively. The effect of durancin GL (200 AU/ml) on the growth of *L. monocytogenes* mutants and the effect on carbohydrate (glucose and mannose) utilization of *L. monocytogenes* mutants were tested as the same method stated above.

### 2.7 Construction of prey and bait vectors

The gene of durancin GL (GenBank: HQ696461.1), Man‐PTS ⅡC, and ⅡD (GenBank: AF397145.1) was designated as GL, ⅡC, and ⅡD, respectively. According to the target gene, primers with two *Sfi* I restriction sites were designed to amplify these target genes. The general PCR system is 50 μl, consisting of 25.0 μl of 2× Fast *Pfu* fly PCR supermix (TransGen, Beijing, China), 1.0 μl of template DNA, 1.0 μl of forward primer, 1.0 μl of reverse primer, and 22.0 μl of ddH_2_O. The primer pairs of target gene GL, ⅡC, and ⅡD were Pr09/Pr11, Pr12/Pr14, and Pr15/Pr17 for bait vectors of pBT3‐SUC and pBT3‐STE, respectively. The primer pairs of target gene GL, ⅡC, and ⅡD were Pr09/Pr10, Pr12/Pr13, and Pr15/Pr16 for prey vector pPR3‐N, respectively. The PCR conditions were as follows: preheating at 98°C for 5 min, 35 cycles of denaturation at 98°C for 30 s, annealing at 55°C for 30 s, extension at 72°C for 10 s (gene GL) or 24 s (gene ⅡC) or 27 s (gene ⅡD), and final extension at 72°C for 10 min.

The amplified fragments of GL, ⅡC, and ⅡD were digested overnight by *Sfi* I enzyme at 50°C. Then, they were linked to the same digested vectors pBT3‐SUC, pBT3‐STE, and pPR3‐N. *Sfi*I enzyme was provided by Thermo Fisher Scientific Co., Ltd. The recombinant vectors were transformed into *E. coli* Top10 competent cells. And the recombinant *E. coli* Top10 was selected on LB agar plate containing ampicillin (100 g/ml). Positive single colony on the plate was selected and verified by colony PCR and sequencing.

### 2.8 Self‐activation test, cytotoxicity test, and intermolecular interaction test

The recombinant plasmids prepared above were transformed into yeast reporter strain NMY51 chemically competent cells (Table 2). The yeast cells were plated on the YPAD (Land Bridge Co. Ltd) agar plate and cultured at 30°C for 4 days. Three colonies of 28 transformed strains (Table 2) were randomly selected from each YPAD plate and diluted with 1 ml sterile water, and then inoculated on SD‐TL agar plate and SD‐TLHA agar plate. SD‐TLHA agar plate contained a certain concentration of 3AT (the final concentration was 0 mM, 5 mM, 10 mM, and 30 mM, respectively).

**Table 2:** Plasmid combinations in yeast transformation

Combinations	Prey plasmid	Bait plasmid	Description
1	pNubG‐Fe65	pTSU2‐APP	positive control
2	pPR3‐N	pTSU2‐APP	negative control
3	pPR3‐N	pBT3‐STE‐GL	self‐activation test detection
4	pPR3‐N	pBT3‐SUC‐GL	self‐activation test
5	pPR3‐N‐GL	pBT3‐STE	self‐activation test
6	pPR3‐N‐GL	pBT3‐SUC	self‐activation test
7	pPR3‐N	pBT3‐STE‐ⅡC	self‐activation test
8	pPR3‐N	pBT3‐SUC‐ⅡC	self‐activation test
9	pPR3‐N‐ⅡC	pBT3‐STE	self‐activation test
10	pPR3‐N‐ⅡC	pBT3‐SUC	self‐activation test
11	pPR3‐N	pBT3‐STE‐ⅡD	self‐activation test
12	pPR3‐N	pBT3‐SUC‐ⅡD	self‐activation test
13	pPR3‐N‐ⅡD	pBT3‐STE	self‐activation test
14	pPR3‐N‐ⅡD	pBT3‐SUC	self‐activation test
15	pOST1‐NubI	pBT3‐STE‐GL	cytotoxicity test
16	pOST1‐NubI	pBT3‐SUC‐GL	cytotoxicity test
17	pOST1‐NubI	pBT3‐STE‐ⅡC	cytotoxicity test
18	pOST1‐NubI	pBT3‐SUC‐ⅡC	cytotoxicity test
19	pOST1‐NubI	pBT3‐STE‐ⅡD	cytotoxicity test
20	pOST1‐NubI	pBT3‐SUC‐ⅡD	cytotoxicity test
21	pPR3‐N‐GL	pBT3‐STE‐ⅡC	interaction test
22	pPR3‐N‐GL	pBT3‐SUC‐ⅡC	interaction test
25	pPR3‐N‐ⅡC	pBT3‐STE‐GL	interaction test
26	pPR3‐N‐ⅡC	pBT3‐SUC‐GL	interaction test
23	pPR3‐N‐GL	pBT3‐STE‐ⅡD	interaction test
24	pPR3‐N‐GL	pBT3‐SUC‐ⅡD	interaction test
27	pPR3‐N‐ⅡD	pBT3‐STE‐GL	interaction test
28	pPR3‐N‐ⅡD	pBT3‐SUC‐GL	interaction test

### 2.9 Determination of β‐galactosidase in transformants

β‐galactosidase in transformants was determined by HTX β‐galactosidase kit (Dualsystems Biotech). The brief steps were as follows: Firstly, the transformant strains were inoculated with 1% (v/v) inoculum in SD‐TL liquid medium and cultured at 30°C with agitation at 250 rpm for 24 hr. Secondly, the supernatant was discarded after centrifugation at 2,500 × *g* for 5 min. Thirdly, in 96‐well plates, 100 μl pyrolysate mixture (mixed by 995 μl one‐step lysis and assay reagent and 5 μl dye stock solution) was added into each reaction and incubated for 90 min, and then, OD_615_ and at last OD_546_ for each well were determined by microplate reader (SpectraMax M2e, Molecular Devices). Each sample was analyzed in triplicate.

## 3 RESULTS

### 3.1 Effect of durancin GL on *Listeria monocytogenes* growth

**Figure 1:**
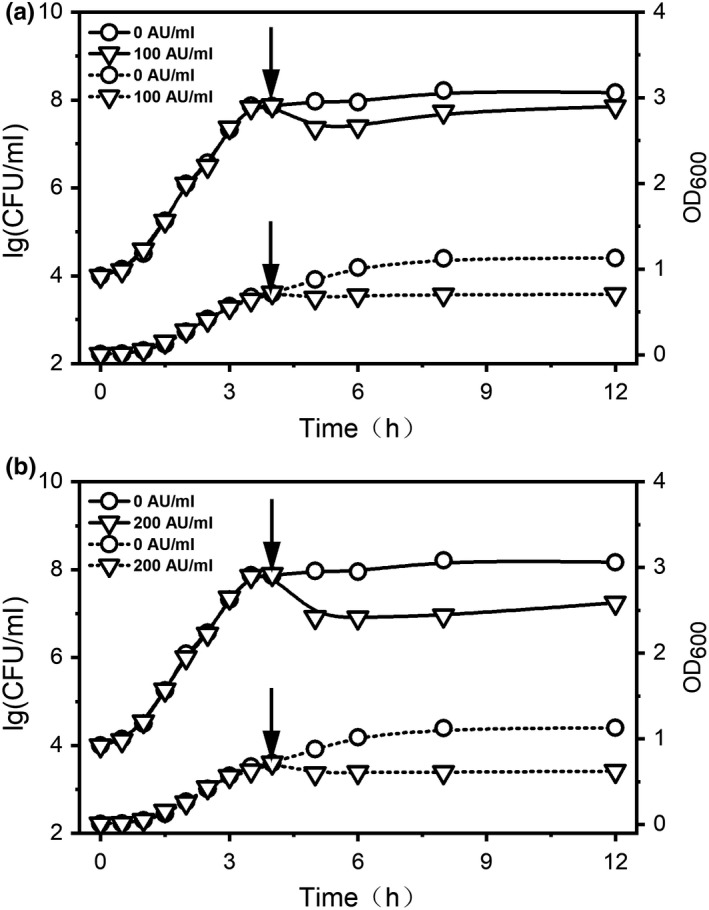
Effect of durancin GL on growth curve of *Listeria monocytogenes*. Solid line is colony plate counting results (represented by logarithmic values), and dotted line is OD_600_. The arrow indicates that a certain amount of bacteriocin durancin GL, the final concentration is 100 AU/ml (a) and 200 AU/ml (b), respectively, is added into the culture system at this time

Previous studies have indicated that durancin GL has a targeted inhibitory effect on *L. monocytogenes* (Du et al., 2012; Ju et al., 2015). This study further confirmed the effect of durancin GL on *L. monocytogenes* growth (Figure 1). During the logarithmic growth period (about 4 hr, OD_600_ ≈ 0.721), the presence of durancin GL suspended the growth of *L. monocytogenes* as the increase of OD_600_ was delayed in the experiment group. The total bacteria (expressed as the logarithm of the total number of colonies) dropped from 8.21 (control group, 0 AU/ml) to 7.73 (experimental group, 100 AU/ml) and 6.96 (experimental group, 200 AU/ml) after treated for 4 hr. The results of bacteria count showed that colonies of *L. monocytogenes* decreased more obviously in experimental group compared with the control group (Figure 1). In high concentration of durancin GL (200 AU/ml, Figure 1b) treatment, the growth of *L. monocytogenes* cells was more restricted than low concentration group (100 AU/ml, Figure 1a). The number of *L. monocytogenes* was strictly controlled for a long time (at least 8 hr) in the durancin GL treatment group.

### 3.2 Effect of durancin GL on carbohydrate utilization by *Listeria monocytogenes*

**Figure 2:**
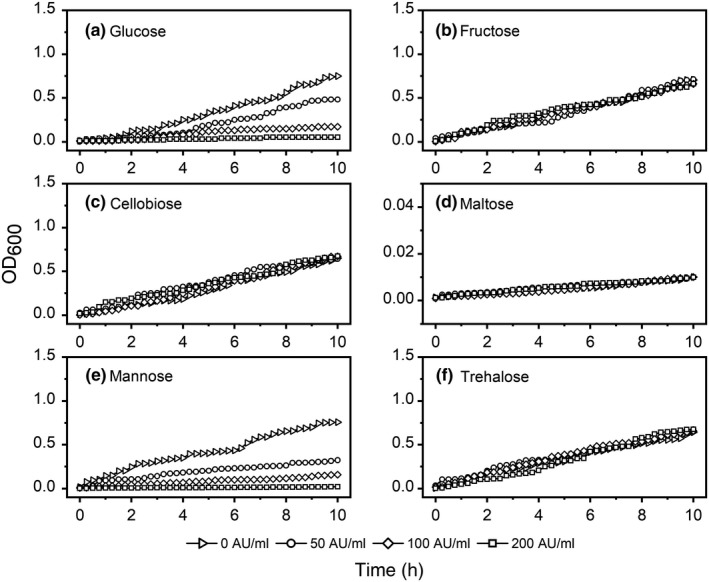
Effect of durancin GL on carbohydrate utilization of *Listeria monocytogenes*. (a) Glucose, (b) fructose, (c) cellobiose, (d) trehalose, (e) mannose, (f) maltose. A certain durancin GL was added to the medium, in which the final concentration was 0, 50, 100, and 200 AU/ml, respectively

Figure 2 shows the effect of durancin GL on the utilization of carbohydrates by *L. monocytogenes*. Based on improved minimum requirement medium, six kinds of sugar were selected as the only source of carbon in this study. The results showed that the presence of durancin GL affected the absorption and utilization of glucose (Figure 2a) and mannose (Figure 2e) by *L. monocytogenes*. After 10 hr, in the compounded glucose medium, the OD_600_ was 0.7500, 0.4825, 0.1700, and 0.0500 for each group (0, 50, 100, and 200 AU/ml). And after 10 hr, in the compounded mannose medium, the OD_600_ was 0.7586, 0.3234, 0.1578, and 0.0196 for each group (0, 50, 100, and 200 AU/ml). However, the absorption and utilization of the other four sugars (Figure 2b, c, d, and f) by *L. monocytogenes* were not affected by durancin GL, regardless of the concentrations (from 0 AU/ml to 200 AU/ml). With the increase of bacteriocin concentration, the absorption and utilization of glucose (Figure 2a) or mannose (Figure 2e) by *L. monocytogenes* were more limited.

### 3.3 Preparation and characterization of *Listeria monocytogenes* mutants

**Figure 3:**
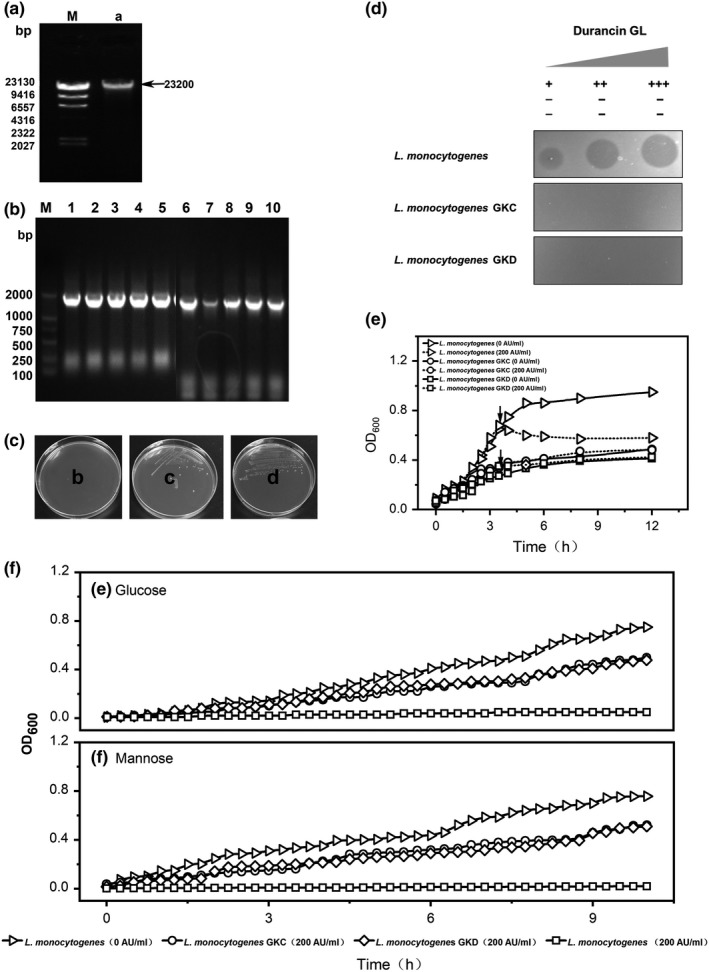
Genetic evidence of bacteriocin durancin GL inhibiting *L. monocytogenes*. (A) Genomic DNA of *Listeria monocytogenes* (line a). M is DNA marker. (B) Identification of colony PCR. M is DNA marker, line 1–5 is *L. monocytogenes* GKC, 6–10 is *L. monocytogenes* GKD. (C) Plate culture of *L. monocytogenes* (b), *L. monocytogenes* GKC (c), and *L. monocytogenes* GKD (d). The plates contain 200 AU/ml durancin GL. (D) Sensitivity of *L. monocytogenes* and its gene mutants to durancin GL. “–” means no antimicrobial zone, “+” means that it has an antimicrobial zone, and the bacteriocin titer is 50 AU/ml; “++” means that it has an antimicrobial zone, and the bacteriocin titer is 100 AU/ml; and “+++” means that it has an antimicrobial zone, and the bacteriocin titer is 200 AU/ml. (E) Growth curves of *L. monocytogenes* and its gene mutants. Solid line is the control group without durancin GL, and dotted line is the experimental group with 200 AU/ml durancin GL. (F) Effect of durancin GL on carbohydrate utilization of *L. monocytogenes* and its gene mutants, (e) glucose, (f) mannose

Genomic DNA of *L. monocytogenes* was extracted, and the result of agarose gel electrophoresis showed it was mainly concentrated in 23 200 bp (Figure 3a). The results were consistent with previous experiments (Wu et al., 2017). After sequencing the knockout vectors, the results regarding to the Man‐PTS ⅡC and Man‐PTS ⅡD knockout boxes (Figure 3b) showed that the corresponding sequences were consistent with the design.

On BHI agar plate containing 50 AU/ml duracin GL, *L. monocytogenes* mutants (*L. monocytogenes* GKC and *L. monocytogenes* GKD) showed visible growth (Figure 3c). Further research showed that *L. monocytogenes* mutants (GKC and GKD) were no longer sensitive to the durancin GL (from 50 AU/ml to 200 AU/ml, Figure 3d). This study further confirmed the effect of durancin GL on growth of *L. monocytogenes* and its mutants (Figure 3e). During the logarithmic growth period, *L. monocytogenes* GKC and *L. monocytogenes* GKD were not affected by the presence of durancin GL (the final concentration was 200 AU/ml).

Figure 3f shows the effect of durancin GL on the utilization of glucose (e) and mannose (f) by *L. monocytogenes* GKC and *L. monocytogenes* GKD. The results indicated that Man‐PTS ⅡC or ⅡD gene knockout resulted in little effect of durancin GL (200 AU/ml) on sugar absorption and utilization of *L. monocytogenes* mutants, indicating that Man‐PTS subunit ⅡC or ⅡD could be related for the sensitivity of *L. monocytogenes* to durancin GL.

### 3.4 Intermolecular interaction between durancin GL and Man‐PTS subunit ⅡC or ⅡD

**Figure 4:**
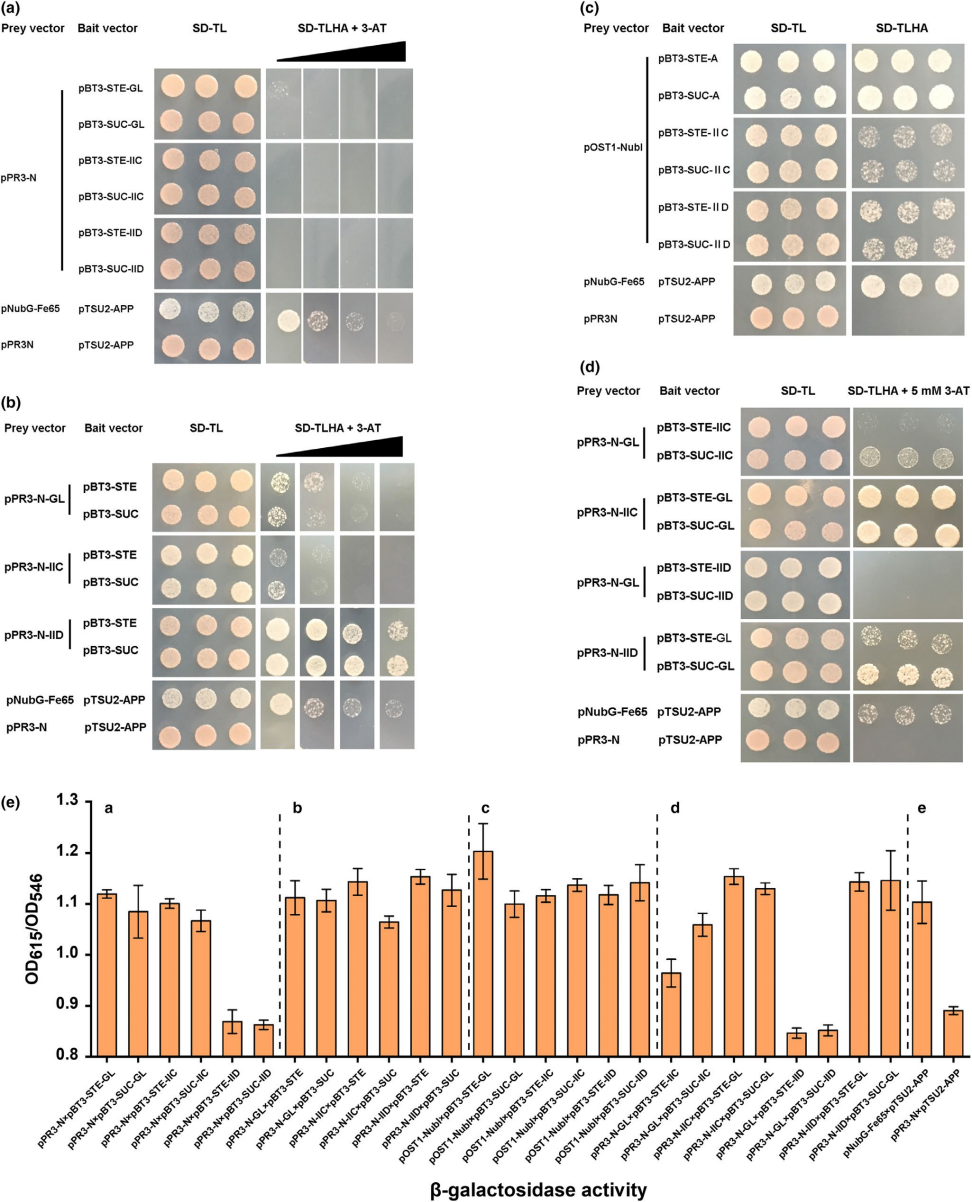
Detection of durancin GL and Man‐PTS ⅡC or ⅡD interaction using yeast two‐hybrid split‐ubiquitin assay. (A) and (B) Self‐activation test of plasmids. The concentration of 3‐AT is 0 mM, 5 mM, 10 mM, and 30 mM, respectively. (C) Cytotoxicity test of plasmids. (D) Intermolecular interaction between durancin GL and Man‐PTS ⅡC or ⅡD. The concentration of 3‐AT is 5 mM. (E) Quantification of β‐gal activity in yeast cells containing various combinations of plasmids. The results are the average from triplicate assays. (a) is corresponding to (A), (b) is corresponding to (B), and so on. (e) is the positive control group (pNubG‐Fe65 × pTSU2‐APP) and negative control group (pPR3‐N × pTSU2‐APP)

To identify the interaction between durancin GL and Man‐PTS subunit ⅡC or ⅡD, we first constructed the yeast two‐hybrid split‐ubiquitin system to detect this interaction. For this purpose, the plasmids listed in Table 2 were constructed, and a pair of plasmids was introduced into yeast reporter strain NMY51, which has two nutritional compensatory reporter genes (*HIS*3, *ADE*2) and a color indicator reporter gene (*Lac*Z). Thus, the interaction between durancin GL and Man‐PTS subunit ⅡC or ⅡD can be determined on the growth of yeast cells on the *SD*‐TL and *SD*‐TLHA plates, as well as *Lac*Z‐dependent color change after incubation in the presence of *Lac*Z substrates (Figure 4).

In order to ensure the accuracy and correctness of intermolecular interaction results, cytotoxicity test and self‐activation test of yeast reporter strain NMY51 with recombinant plasmids were verified (Figure 4a, 4, and 4). Cytotoxicity test showed that all the six bait vectors constructed (pBT3‐STE‐GL, pBT3‐SUC‐GL, pBT3‐STE‐ⅡC, pBT3‐SUC‐ⅡC, pBT3‐STE‐ⅡD, and pBT3‐SUC‐ⅡD, in Table 2) can be expressed smoothly in yeast cells (Figure 4c), and *Lac*Z reporter gene was activated with β‐galactosidase activity was much higher than the negative control group (Figure 4c and e). However, there is little self‐activation for target gene (gene GL, ⅡC, and ⅡD) located in bait vector (Figure 4b, Figure 4b). As we all know, a certain concentration of 3‐AT could eliminate some background growth on the selection plates (Vidalain, Boxem, Ge, Li, & Vidal, 2004), and thus, it was necessary to add a certain concentration of 3‐aminotriazole (3‐AT, from 0 mM to 30 mM) for inhibiting self‐activation (Figure 4a and 4). The results showed that 5 mM 3‐AT was sufficient to inhibit the self‐activation above.

As the positive control, yeast reporter strain NMY51 with vector pNubG‐Fe65 and pTSU2‐APP grew well on SD‐TL and SD‐TLHA plates and showed strong β‐galactosidase activity (OD_615_/OD_546_ = 1.103) because of the interaction of expressed cytosolic protein Fe65 (amyloid beta A4 precursor protein‐binding family B member 1) and APP (amyloid A4 precursor protein), but yeast reporter strain NMY51 with vector pPR3‐N and pTSU2‐APP did not grow on SD‐TLHA plates and showed little β‐galactosidase activity (OD_615_/OD_546_ = 0.890), which was used as a negative control. The results showed that yeast reporter strain NMY51 with vector pPR3‐N‐GL and pBT3‐SUC‐ⅡC or pPR3‐N‐ⅡC and pBT3‐STE‐GL or pPR3‐N‐ⅡC and pBT3‐SUC‐GL grew well on the SD‐TL and SD‐TLHA plates and showed a higher β‐galactosidase activity than negative control, which OD_615_/OD_546_ was 1.059, 1.153, and 1.129, respectively; however, yeast reporter strain NMY51 with vector pPR3‐N‐GL and pBT3‐STE‐ⅡD or pPR3‐N‐GL and pBT3‐SUC‐ⅡD did not grow on the SD‐TLHA plates, and yeast reporter strain NMY51 with vector pPR3‐N‐ⅡD and pBT3‐STE‐GL or pPR3‐N‐ⅡD and pBT3‐SUC‐GL grew well same as the self‐activation of pPR3‐N‐ⅡD (Figure 4b). Based on the above experimental results, it has indicated that there was a strong interaction between durancin GL and Man‐PTS subunit IIC. However, the interaction between durancin GL and Man‐PTS subunit IID was not present or weak.

## 4 DISCUSSION

In order to clarify the targeting inhibition of *L. monocytogenes* by durancin GL, a new class Ⅱa bacteriocin, *L. monocytogenes* mutants with Man‐PTS IIC or IID deletion were constructed; then, its utilization of glucose and mannose and the sensitivity to durancin GL were investigated, and in further, the interactions between durancin GL and the subunits of IIC or IID in Man‐PTS of *L. monocytogenes* were characterized by yeast two‐hybrid split‐ubiquitin system. The genetic and biochemical evidence provided in this study revealed that Man‐PTS subunit IIC was the target/receptor for sensitivity of *L. monocytogenes* to durancin GL. Man‐PTS subunit IIC combined with durancin GL to form some kind of complex, which limited the carbohydrate utilization of glucose and mannose, thereby inhibiting the growth and reproduction of *L. monocytogenes*.

By monitoring the growth dynamics (Wu, Yin, Hsu, & Jiang, 2005), the inhibitory effect of pediocin ACCEL (class IIa, pediocin‐like bacteriocins) on *L. monocytogenes* was similar to that in this study. Similar results of antilisterial bacteriocins have been reported in several studies involving bacteriocins of bactofencin A (O'Shea et al., 2013), plantaricin LPL‐1(Wang, Qin, Zhang, Wu, & Li, 2018), lacticin 3147 (Deegan, Cotter, Hill, & Ross, 2006), Apb 118(Corr et al., 2007), and sakacin P (Tessema, Moretro, Kohler, Axelsson, & Naterstad, 2009). Besides, the effect of mesentericin Y105, a class Ⅱa bacteriocin, on four sugar utilization by sensitive bacteria showed that the sensitivity of *E. faecalis* was highly increased in the presence of glucose or mannose, compared to cellobiose or fructose (Hechard, Pelletier, Cenatiempo, & Frere, 2001), and the sensitivity of *L. monocytogenes* was affected in a medium supplemented with mannose or glucose but not with cellobiose or fructose (Karine, Yves, Pascale, & Yann, 2001). Many reports about Man‐PTS components as bacteriocin receptors have been proved experimentally. It was reported that Lactococcin A affected on the carbohydrate utilization by *L. monocytogenes*, and the correlation between ptn gene and Lactococcin A sensitivity was verified by gene knockout and gene replacement experiments (Diep et al., 2007). It was an observation further supporting the notion that the Man‐PTS components act as a receptor targeting for bacteriocin. To gain insight into bacteriocin resistance, four class IIa bacteriocin (pediocin PA‐1) resistant mutants of *E. faecalis* were obtained, and further research data confirmed the important role of Man‐PTS in class IIa bacteriocin sensitivity and we demonstrate its importance involving global carbon catabolite control (Opsata et al., 2010). Stoll and Goebel, (2010) examine the major PEP‐dependent phosphotransferase systems of *L. monocytogenes* by a systematic deletion analysis and identified the major PTSs involved in glucose, mannose, and cellobiose transport. A growing number of reports suggest that Man‐PTS components act as a receptor targeting for bacteriocin (Colombo et al., 2018). Besides, several researches revealed that the Man‐PTS subunit ⅡD also was target/receptor for the sensitivity of target bacteria on bacteriocin (Stoll & Goebel, 2010; Tymoszewska, Diep, & Aleksandrzak‐Piekarczyk, 2018; Zhou et al., 2016). However, credible evidence was not observed in this study because of interaction between durancin GL and Man‐PTS subunit IID was not present or weak.

Although several plasmids have been used for *L. monocytogenes* generating mutants by allelic exchange, construction of *L. monocytogenes* mutants has been inefficient due to lack of effective selection markers for first and second recombination events (Abdelhamed, Lawrence, & Karsi, 2015). A new suicide plasmid pHoss1 provides answers to the above questions. Gene knockout vector pHoss1 was constructed by Hossam et al. (Abdelhamed et al., 2015), which have successfully knocked out some genes of *L. monocytogenes*, and the success rate is 80%–100%. Based on the principle of homologous recombination, Man‐PTS component ⅡC‐ and ⅡD‐related genes of *L. monocytogenes* were knocked out with the help of vector pHoss1, and the mutants of *L. monocytogenes* with deletions of Man‐PTS ⅡC and ⅡD were constructed in this study.

Despite its great popularity, the greatest disadvantage of the classical yeast two‐hybrid system is the obligatory nuclear localization of the proteins and, hence, their site of interaction. Five years after the initial description of the yeast two‐hybrid system, an alternative with the potential to overcome these limitations was described. The split‐ubiquitin system never managed to gain as much popularity as the yeast two‐hybrid system despite providing the same ease of application, yet it allows virtually all protein types to be tested without the need to truncate or mislocalize these proteins and without introducing additional artifacts to those associated with the yeast two‐hybrid system (Xing, Wallmeroth, Berendzen, & Grefen, 2016). The interaction between bacteriocin durancin GL and Man‐PTS ⅡC or ⅡD was detected by yeast two‐hybrid split‐ubiquitin system. Although both ⅡC and ⅡD are components of Man‐PTS, they interact with bacteriocins durancin GL in the opposite way. There are many possible reasons for this. Firstly, there is no interaction between bacteriocin durancin GL and Man‐PTS IID. Secondly, because of the structural differences between Man‐PTS ⅡC and Man‐PTS ⅡD, the former has 7 transmembrane regions, and the latter has four transmembrane regions. Thirdly, it may also be related to the location of membrane subunit in yeast cells. All of the above may need further confirmation in future research.

Although this study has revealed the mechanism of bacteriocins durancin GL inhibiting *L. monocytogenes*, there are still a lot of unclear aspects in its physical and chemical properties, bacteriostasis process and related mechanism and so on. As a new bacteriocin, clarifying the relevant mechanism of durancin GL inhibiting *L. monocytogenes* is not only provides a new choice for the development of efficient natural food preservatives, but also provides a scientific basis for further clarifying the role of bacteriocins.

## 5 CONCLUSION

In this study, results indicated that durancin GL, a new bacteriocin, had has obvious antibacterial activity against *L. monocytogenes*. The presence of durancin GL affected the absorption and utilization of glucose and mannose by *L. monocytogenes*; however, the *L. monocytogenes* Man‐PTS gene mutants exposed to bacteriocin durancin GL still grow and utilize glucose and mannose in the medium. Furthermore, an obvious intermolecular interaction between durancin GL and Man‐PTS subunit ⅡC is confirmed in this report. This provides a basis for sensitivity of *L. monocytogenes* to durancin GL.

## CONFLICT OF INTEREST

The authors declare that they have no conflict of interest.

## ETHICAL APPROVAL

The study did not involve any human or animal testing.

## INFORMED CONSENT

Written informed consent was obtained from all study participants.
